# Comparing Free Play and Partly Structured Play in 4-5-Years-Old Children in an Outdoor Playground

**DOI:** 10.3389/fpubh.2019.00197

**Published:** 2019-07-16

**Authors:** Patrizia Tortella, Monika Haga, Jan Erik Ingebrigtsen, Guido Francesco Fumagalli, Hermundur Sigmundsson

**Affiliations:** ^1^Faculty of Education, University of Bozen, Bolzano, Italy; ^2^Section for Arts, Physical Education, and Sports, Department of Teacher Education, Norwegian University of Science and Technology, Trondheim, Norway; ^3^Department of Sociology and Political Science, Norwegian University of Science and Technology, Trondheim, Norway; ^4^Public Health and Community Medicine, Research Centre on Child Motor Development, University of Verona, Verona, Italy; ^5^Associazione Onlus Laboratorio, Treviso, Italy; ^6^Department of Psychology, Norwegian University of Science and Technology, Trondheim, Norway; ^7^Sports Science Department, Reykjavik University, Reykjavik, Iceland

**Keywords:** motor development, movement skills, outdoor play, organization, kindergarten

## Abstract

The aim of this study was to compare how the organization of a movement session as partly structured play or free play influenced the physical activity engagement in 4–5 years old pre-schoolers. The partly structured playgroup consisted of 46 children and the free playgroup consisted of 33 children. The playground activities consisted of 10 sessions each lasting 1 h, executed once per week in the period Mars to May 2017 at a specific playground setting. The partly structured playgroup conducted a movement activity session that included a combination of both structured- and free play activities. The free playgroup engaged in unstructured play, only. To detect the intensity of the physical activity each child carried an accelerometer 1 h the first week and last week of the intervention. Results indicate a significant difference in physical activity level between the two groups for the 5-year-old in the favor of the partly structured playgroup. There was a significant difference between the four-and 5-year-old in relation to physical activity level. No significant difference between the activity in March and May for the whole group was found.

## Introduction

Engagement in physical activity from a young age is recognized as a critically important factor for good physical and mental health. Specifically, participation in physical activity is linked to healthy weight status, motor skill development, psychosocial health and metabolic health indicators in preschoolers (1–4). To gain the maximum benefits associated with physical activity, recommendations have been generated regarding the amount of physical activity needed daily in this population. More recently, these 24-h movement guidelines have accredited that the whole day matters and individual movement behaviors like physical activity, sedentary behavior and sleep must be considered in relation to each other when exploring their associations to health indicators and developmental outcomes (5, 6). Guidelines recommend that preschoolers (accumulate at least 180 min of daily physical activity at any intensity, which at least 60 min is energetic play (e.g., moderate- to vigorous-intensity physical activity). Additionally, children at this age should not be restrained for more than 1 h at a time or sit for extended periods of time and sedentary screen time should be no more than 1 h (5, 6). The reciprocal association between physical activity and motor development across childhood should also be addressed (7). The type and intensity of physical activity will lead to both quantitative and qualitative development of motor skill, that is, development and learning of new motor skills and refinement/improvement of already learned motor skills (8). Given that healthy habits and behavior, including physical activity and sedentary behavior, acquired in childhood typically track into later life (9, 10), these recommendations are especially important for health outcomes across the lifespan (11).

Driven by the health and psychological concerns related to lack of physical activity, several studies have investigated the behavior of preschool children in various environmental contexts. In Western societies, a common finding is that physical activity levels are lower than recommended (12, 13). A Norwegian study reports that 32 percent of the girls and 67% of the boys reached the recommended level of 60 min of moderate to vigorous physical activity per day during their time in preschool (14).

A wide range of barriers and facilitators to young children's physical activity and sedentary behavior are put forward. For both behaviors a broad range of potential correlates are found, including demographic, biological, environmental, social, and psychological factors (15). Moreover, both the indoor and the outdoor environments (e.g., equipment, organization, urban/rural areas) and their organization influence upon the physical activity levels of preschool children (16–19). In this context, an under-researched area is how the organization, structure and purpose of the movement activity may influence physical activity engagement in this population (20). Active, or free, play is defined as “a form of gross motor or total body movement in which young children exert energy in a freely chosen, fun, and unstructured manner” [(21), p 164]. This term can be placed into the framework of deliberate play (22), which is characterized as “a form of sporting activity that involves early developmental physical activities that are intrinsically motivating, provide immediate gratification, and are specifically designed to maximize enjoyment” [(22), p 185–186]. On the other hand, structured movement sessions or “*deliberate practice* is planned and designed (a) with the specific purpose of increasing performance, (b) requiring cognitive and/or physical effort, and (c) relevant to promoting positive skill development” [(22), p 185]. The differences in physical activity engagement between free (unstructured) and structured play sessions are not well understood in young children and weather one has a larger potential to influence outcomes such as physical activity, motor competence and obesity is unclear (23–25). Thus, a better knowledge of how specific programs and interventions can promote physical activity and motor development in early childhood is required.

Johnstone et al. (25) included five papers in their systematic review on active play interventions as promotion of physical activity and fundamental motor skills among pre-schoolers. Two studies suggest that free play may be a potential useful approach to increase the total amount of physical activity (26, 27). Free play was also found to generate additional benefits beyond increasing physical activity levels, including improved gross motor skills (28). In sum, Johnstone et al. (25) conclude that due to the small number of eligible studies no conclusion on the effect of free play interventions on pre-schoolers engagement in physical activity can be drawn. In contrast, there is some evidence to suggest that structured play appears to be more efficient than free play in producing high physical engagement during play (20, 29). Similar, research has shown that structured programs were effective in improving and maintaining motor skills (30, 31).

The purpose of this study was to examine differences in physical activity engagement between a partly structured- and a free (unstructured) movement session executed at the same outdoor playground setting in 4–5 years old children. The following research questions were set out;

Is there difference in physical activity level (counts per minute) between the partly structured playgroup and the free playgroup and is there any change in physical activity level (counts per minute) after the intervention period, i.e., from March to May?

## Materials and Methods

### Participants

Four out of 12 kindergartens in Treviso, Veneto, northern Italy were selected for participation in the study based on similarities of the socio-economic status and ethnic origin of the families. Hundred and seven children were recruited to the study, out of these, 79 children completed the measurement at pre- and posttest. Two kindergartens participated in the partly structured playgroup conducting a movement activity session that included both structured and free play activities at the playground consisting of 19 children aged 4 years (4 boys and 15 girls; mean age 4.53 ± 2.7) and 27 children aged 5 years (15 boys and 12 girls, mean age 5.63 ± 0.3). Children from two other kindergartens conducted a session of free play. The group of 4 years old consisted of 14 children (7 boys and 7 girls, mean age 4.45 ± 0.18) and the 5-year-old consisted of 19 children (13 girls and 6 boys, mean age 5.61 ± 0.33).

### Procedures

The study was approved by the Ethics and Scientific Committee of Laboratorio 0246, Treviso, Italy, the non-profit Association that owned the site where the research was carried out. The Committee verified the appropriateness of the documentation and procedures and verified the adherence to the principles of the Declaration of Helsinki. Written informed consents were obtained from the parents (or guardians) before the children attended the study. All parents received extensive written descriptions of the goals, limits and risks of the study, of the methods used and of the activities performed before being asked to sign authorizations. Field permission of the study was granted by: ASD Laboratorio 0246 no-profit—Strada del Nascimben 1/B 31100 Treviso Phone: +39 0422 324310 Fax: +39 0422 324311 Email: info@0246.it; http://www.0246.it/.

### The Primo Sport 0246 Playground

**Figure 1:**
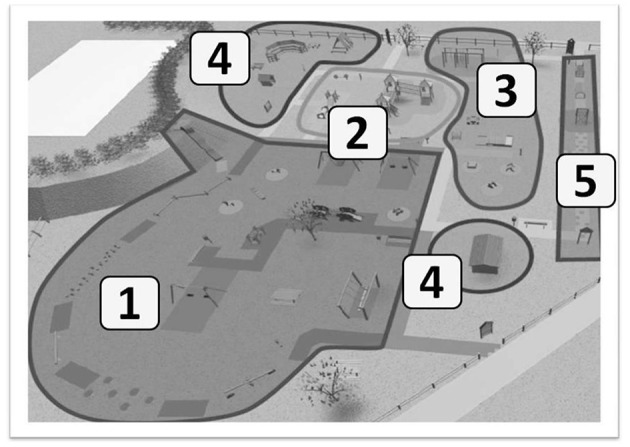
General layout out of the playground Primo Sport 0246 and distribution of the specific areas. 1. Balance area; 2. Mobility area; 3. Manuality area; 4. Symbolic play area; 5. Mixed area.

The playground Primo Sport 0246 (see Figure 1) is a private playground but is open to public. The playground is located in Treviso and was designed to provide controlled opportunities for practicing basic motor skills to children from 0 to 6 years (32).

### Main Features of the Playground Are the Following

The playground has a size of 2500 m2 and contains a total of 35 playground equipment and instruments that are placed at a safe distance from each other. The playground surface consists of different consistency/softness to limit any injuries in case of falls and accidents. The playground is divided in five dedicated areas where instruments are located based on the motor skill most trained by their use (see Figure 1). In the manual dexterity, mobility and balance areas, instruments were selected to provide different levels of difficulties so that each child could exercise the skill regardless of his/her own level of competence [for detailed description of the organization of the park seeBuzzavo et al. (32)].

### Organization of Activities at the Playground

An objective measurement of physical activity (accelerometer) was applied in the first and last session in an activity period consisting of 10 visits to a specific playground during a period of 10 weeks in the period Mars to May 2017. During each visit, occurring once per week, one group of children were exposed to both structured- and free play (partly structured playgroup) (30 min in each of them) and the other group of children participated in 60 min of free play (unstructured playgroup), only. Both groups conducted the movement session in the same outdoor playground with the same facilities and equipment available (see description of The Primo Sport 0246 playground). For the purposes of this study, a structured play session is defined as a planned movement time designed to incorporate opportunities to practicing basic motor skills and make use of large muscle groups.

All sessions took place between 9 am and 12 am, with temperatures ranging between 10 and 26 degrees Celsius; there were no raining days during the study. The two groups went to the playground at different days.

## The Group of Partly Structured Activity

**Figure 2:**
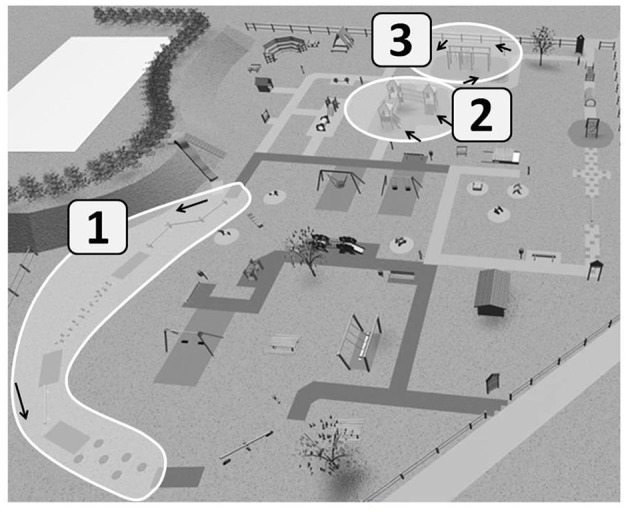
Whitened areas show where structured activities were run. 1. Balance area; 2. Mobility area; 3. Manuality area. Active play (unstructured activities) could be done everywhere else in the park at free choice of the child. Total time for structured paly was 30 min (10 min in each of the whitened areas) and time for active play was also 30 min.

The session of partly structured play lasted for 60 min in total, 30 min in structured activity and 30 min in free play. The group was divided in two, one half started with free play and the other half started with structured activity. After 30 min the groups switched type of activity, e.g., from structured play to free play or vice versa. The children that began with structured activity were further divided in three small groups of 6–7 children with each subgroup spending 10 min in portions of each of the three dedicated areas (see Figure 2). The sequence of the activities was: (1) Manual dexterity: the children stayed on a tool for about 30 s before turning to the next tool; rope ladder, climbing rope, hanging bar, gymnastic rings, climbing net, monkey bars, spending in total 10 min in this area. (2) Balance: the children played on the following tools in the sequence: balance beam, balance logs, balance elastic beam, balance platforms. The children repeated the circle about three times. (3) Mobility: each child went up and down from various climbing points and slopes in an organized sequence.

One instructor, trained to the program, was constantly present in the manual dexterity area, one in the mobility area and one in the balance area. The instructors were trained to provide scaffolding if requested, give instructions about possible ways of use of instruments and provided general encouragement for exploring the various aspects and challenges associated to each of the playground activities. Another instructor controlled the time spent in each area and coordinated the switch of the groups from one area to the other. Free play was allowed everywhere within the playground, except for the portions of the areas where the other group was performing the structured activities. Kindergarten teachers (at least one every 10 children) were present for assistance and supervision, without involving in the activities (no instructions, guiding or encouragement).

## The Group of Free Play

Free play was allowed everywhere within the playground for 60 min. One instructor, trained to the program, was constantly present in playground to control time. Kindergarten teachers (at least one on every 10 children) were present for assistance and in case of emergency but did not involve in the activities (no instructions, guiding or encouragement).

### Assessment of Physical Activity (Accelerometer)

To detect the intensity of the physical activity engagement at the playground, the children wore ActiGraph GT3X, with an elastic belt on the right hip. The monitors connected data at the vertical ax, in 60 s intervals (epochs) and the output of the ActiGraph is given in counts pr. minute (cpm). The counts obtained during the time at the playground are related to the average intensity of the children's activity through the time at the playground. According to the cut-offs set by Pate et al. (33), moderate physical activity was identified at 1,680 cpm, and any amount above 3,368 cpm was considered vigorous intensity physical activity. There was no missing data during recording and downloading the accelerometers.

### Data Reduction and Analysis

The data were analyzed in SPSS (version 15) and statistical significance was set at *P* < 0.05. Non-parametric statistic was used, Mann-Whitney *U*-test and Wilcoxon Rank test.

## Results

### Partly Structured Play vs. Free Play

Table 1 shows the results from the accelerometer for the two data points in March and May for the two groups participating in partly structured play or free play, only. There were no significant differences in cpm between partly structured play vs. free play for the whole sample (4 and 5 years) (Mann-Whitney *U*-test: Z = −1.710, *p* = ns). Neither was there any significant difference in the group of 4 years old (Mann Whitney *U*-test Z = −0.801, *p* = ns). In the 5-year-old, the difference in cpm was low but significant (Mann Whitney *U*-test Z = −2.019, *p* = 0.04).

**Table 1:** Physical activity level measured as counts per minutes (cpm) in structured/active play and active play for 4 and 5 years old (for the two data points in March and May together).

	**Structured/active play**	**Active play**	P^*^
	**CPM Mean (*SD*)**	**CPM Mean (*SD*)**	
4 and 5-year-old	2427 (963)	2002 (585)	ns.
March	2504 (1186)	1915 (672)	0.003
May	2221 (892)	2101 (755)	ns.
4-year-old	1874 (487)	1714 (515)	ns.
March	2091 (540)	1619 (656)	0.003
May	1615 (508)	1879 (514)	ns.
5-year-old	2816 (1029)	2213 (552)	0.04
March	2788 (1415)	2152 (600)	ns.
May	2671 (852)	2245 (857)	0.038

* *Mann-Whitney U-test*.

Table 2 show the data for the accelerometer for the 4-year-old and 5-year-old, separately. There was a significant difference between the 4-year-old and 5-year-old in relation to cpm (Mann Whitney *U*-test Z = −4.294, *p* < 0.001).

**Table 2:** Physical activity level measured as counts per minutes (cpm) for the 4 and 5-year-old children (for the two data points in March and May).

	**4-year-old**	**5-year-old**	P^*^
	**Mean (*SD*)**	**Mean (*SD*)**	
Counts per minute	1806 (498)	2406 (940)	<0.001

* *Mann-Whitney U-test*.

### Accelerometer Data for March vs. May

Table 3 indicate the difference between counts per minute in March vs. May.

**Table 3:** Physical activity level measured as counts per minutes (cpm) in March vs. May for the whole sample (*n* = 79).

	**March**	**May**	P^*^
Cpm (The whole sample)	2230 (1092)	2134 (820)	ns.
Structured/active play (cpm)	2504 (1186)	2221 (892)0.029	0.029
Active play (cpm)	1915 (673)	2101 (755)	ns.

* *Wilcoxon Ranks test*.

There was not a significant difference between the activity in March and May (Wilcoxon Signed Ranks Test Z = −0.828, *p* = ns).

## Discussion

As noted earlier, a wide range of facilitators and barriers to physical activity behavior in young children has been identified (15, 34). The present study compared how the organization of the movement session as partly structured play or free play influenced the physical activity engagement in 4–5 years old children.

### Physical Activity Levels in Partly Structured Play vs. Free Play

There were no significant differences in cpm between partly structured play vs. free play for the whole sample (4 and 5 years) or in the group of 4 years old (see Table 1). During the movement activity session in the playground, the children had an average activity level of moderate intensity, regardless of belonging to the partly structured playgroup or the free playgroup. In this age group, slow walk gives about 1,500 cpm, brisk walk gives nearly 3,000 cpm and jogging about 4,000 cpm. Giske et al. (35) found in their study of 5-year-old children in a day care center, that the average physical activity in outdoor play was about 1,300 cpm. The partly structured playgroup was mainly engaged in movement tasks such as balance and manual dexterity in their 30 min of structured play. If the structured activity had been organized with other activities of higher intensity, such as running or jumping, this could have influenced the activity levels measured as cpm. Thus, the type of activity, which is structured (e.g., climbing or running), will by itself influence the participants activity level. In the 5-year-old, the difference in cpm was low but significant in favor of the group who engaged in partly structured play. It can be suggested that 5 years old children have more benefit from scaffolding, instructions, and general encouragement for exploring the various aspects and challenges associated to each of the playground activities compared to the younger children. Older children may try to perform more advanced and challenging motor actions than younger children do. In this way, they will benefit more from encouragement, instruction and individual feedback on their movement performances in order to master the activity and hence, maintain a higher activity level. In line with this, there is evidence that structured activities improve motor skill development in the intervention group compared to the control (28, 36). Another explanation could also be that the combination of structured and free play facilitated children to maintain a higher engagement in activity during the session of 60 min due to instructions and motivation from the instructors. As observed under the intervention, some of the children in the free playgroup were more active in the beginning of the movement session but did not keep the same level of activity during the whole session. As it was a higher activity engagement among the 5 years old children participating in partly structured playgroup but no effect of group in the youngest children (4 years) this finding may suggest that organization of play sessions (free or structured) may have various effect at different ages.

Playground environment such as play equipment, playground markings, play space and localization (i.e., indoor or outdoor) are found to influence physical activity engagement in preschoolers (37, 38). However, some studies have not considered the possible environmental influence when comparing different types of physical activity play. For example, in addition to different play interventions the groups were also exposed to different settings such as indoor or outdoor localization [see for example Palmer et al. (20)]. In our study both groups had access to the same playground settings and the findings of missing or small difference in activity engagement between groups could indicate that for preschoolers, enough time and space to play is sufficient to be physically active (38).

There was significant difference between the 4-year-old and 5-year-old in relation to counts per minute (see Table 2). This is supported by findings of increased level of total physical activity and more time spent in moderate to vigorous activity with increasing age (from 3 to 6 years) (39, 40). Forms of physical activity play include for example gross locomotor movements (like running, climbing, and chasing) that likely peak around 4 to 5 years (41, 42). Thus, a plausible explanation could be that advancing motor competence in the oldest children contributes to an increase in physical activity level (43). Physical activity seems to peak around the age of five and decrease cross-sectionally each year after age 5, with a corresponding increase in time spent sedentary (39).

### Physical Activity Levels in May vs. March

Regarding the change in physical activity level after the intervention period, there was no significant difference between the activity level in May vs. March for the whole sample, suggesting no increase in physical activity after a period of 10 sessions of movement activity organized as partly structured play or free play, respectively (see Table 3). However, children in the partly structured playgroup displayed significant higher cpm in the beginning of the intervention period compared to at the end. Decreasing motivation to engage into physical activity play at the same playground over time could possibly explain this decrease. In addition, this group could engage in the same motor skills as in the structured play during the free playtime. For example, experience to play at the balance beam could cause increased time spent in this task, a task that is an activity requiring more precise and accurate coordination than high intensity.

In the context of children's free play and sport, the nature of childhood has changed over recent generations and there is a common concern that children no longer play the way previous generations did (44). For many children, structured movement programs may be the only opportunity to engage in physical activity throughout the day (30). Structured play with skilled instructor has an advantage as they can facilitate and adapt activity to the individual level of performance, i.e., giving each child right challenge in relation to skill proficiency. However, interventions attempting to change participation in organized physical activity have small effect on total physical activity engagement (45). On the other hand, free play has the potential to be endorsed in a variety of settings (25) and is found to increase total physical activity engagement in younger children (26, 27). Moreover, to determine the potential of play is further complicated by the lack of a clear definition of different play activities and subsequent variability in how physical activity play is measured (21).

We acknowledge that this study may have some limitations. Although the use of accelerometer is an objective measure of children's physical activities, accelerometers could underestimate some physical activity, e.g., are unable to quantify activities like upper-body activities. On the other hand, accelerometer can also overestimate activities that is of relatively low intensity such as playing on a swing. In this intervention, it can be considered as a strength that both groups had access to the same playground setting and controlling for the possible effect of environmental conditions such as location (indoor/outdoor), equipment and space.

In general, results indicate a tendency for a higher physical activity level in the partly structured playgroup compared to the free playgroup. However, there were only a significant difference in physical activity level between the two groups for the 5-year-old in the favor of the partly structured playgroup. As expected, there was significant difference in counts per minute between the 4-year-old and 5-year-old, in favor of the 5 years old. No change in physical activity level was found between March and May for the whole sample, however, children in the partly structured playgroup had significant higher cpm in the beginning of the intervention compared to at the end. During the movement activity session in the playground, the children had an average activity level of moderate intensity, regardless of whether they belonged to one or the other group. Guidelines recommend that preschoolers accumulate at least 180 min of daily physical activity at any intensity, which at least 60 min is energetic play (e.g., moderate- to vigorous-intensity physical activity). In this context, enriched environments, such as playgrounds, could promote physical activity engagement and make children able to meet the recommended movement guidelines.

## Data Availability

All datasets generated for this study are included in the manuscript and/or the supplementary files.

## Ethics Statement

At the time of the study, an ethics approval was not required by the University of Verona which did not have an Institutional Review Board for studies that were not involving patients of the University Hospital. Accordingly, the project was examined by the Ethics and Scientific Committee of Laboratorio 0246, the non-profit Association that owned the site where the research was done and has organized the activities with the schools. The Committee verified the appropriateness of the documentation and procedures and verified the adherence to the principles of the Declaration of Helsinki. Written informed consents were obtained from the parents (or guardians) before the children attended the study.

## Author Contributions

PT: experiment planning and organization, data acquisition, and text writing. MH and GF: experiment planning and organization, data analysis, and text writing. JI: experiment planning and organization, data acquisition, and data analysis. HS: experiment planning and organization, data acquisition data analysis, and text writing.

### Conflict of Interest Statement

The authors declare that the research was conducted in the absence of any commercial or financial relationships that could be construed as a potential conflict of interest.
